# Epigenetic age is associated with baseline and 3-year change in frailty in the Canadian Longitudinal Study on Aging

**DOI:** 10.1186/s13148-021-01150-1

**Published:** 2021-08-23

**Authors:** Chris P. Verschoor, David T. S. Lin, Michael S. Kobor, Oxana Mian, Jinhui Ma, Guillaume Pare, Gustavo Ybazeta

**Affiliations:** 1 Health Sciences North Research Institute, 41 Ramsey Lake Road, Sudbury, ON P3E 5J1 Canada; 2 Northern Ontario School of Medicine, Sudbury, ON Canada; 3 Department of Health Research Methods, Evidence and Impact, McMaster University, Hamilton, ON Canada; 4 BC Children’s Hospital Research Institute, Centre for Molecular Medicine and Therapeutics, University of British Columbia, Vancouver, BC Canada

**Keywords:** Frailty, Epigenetic clock, DNA methylation, CLSA

## Abstract

**Background:**

The trajectory of frailty in older adults is important to public health; therefore, markers that may help predict this and other important outcomes could be beneficial. Epigenetic clocks have been developed and are associated with various health-related outcomes and sociodemographic factors, but associations with frailty are poorly described. Further, it is uncertain whether newer generations of epigenetic clocks, trained on variables other than chronological age, would be more strongly associated with frailty than earlier developed clocks. Using data from the Canadian Longitudinal Study on Aging (CLSA), we tested the hypothesis that clocks trained on phenotypic markers of health or mortality (i.e., Dunedin PoAm, GrimAge, PhenoAge and Zhang in Nat Commun 8:14617, 2017) would best predict changes in a 76-item frailty index (FI) over a 3-year interval, as compared to clocks trained on chronological age (i.e., Hannum in Mol Cell 49:359–367, 2013, Horvath in Genome Biol 14:R115, 2013, Lin in Aging 8:394–401, 2016, and Yang Genome Biol 17:205, 2016).

**Results:**

We show that in 1446 participants, phenotype/mortality-trained clocks outperformed age-trained clocks with regard to the association with baseline frailty (mean = 0.141, SD = 0.075), the greatest of which is GrimAge, where a 1-SD increase in ΔGrimAge (i.e., the difference from chronological age) was associated with a 0.020 increase in frailty (95% CI 0.016, 0.024), or ~ 27% relative to the SD in frailty. Only GrimAge and Hannum (Mol Cell 49:359–367, 2013) were significantly associated with change in frailty over time, where a 1-SD increase in ΔGrimAge and ΔHannum 2013 was associated with a 0.0030 (95% CI 0.0007, 0.0050) and 0.0028 (95% CI 0.0007, 0.0050) increase over 3 years, respectively, or ~ 7% relative to the SD in frailty change.

**Conclusion:**

Both prevalence and change in frailty are associated with increased epigenetic age. However, not all clocks are equally sensitive to these outcomes and depend on their underlying relationship with chronological age, healthspan and lifespan. Certain clocks were significantly associated with relatively short-term changes in frailty, thereby supporting their utility in initiatives and interventions to promote healthy aging.

**Supplementary Information:**

The online version contains supplementary material available at 10.1186/s13148-021-01150-1.

## Background

Over the past 20 years, there has been a great intensity of research into tools and methods available to quantify the health trajectories of older adults and, more importantly, classify their vulnerability to adverse outcomes. The concept of “biological age,” which increases with accumulated damage (or wear and tear) caused by both acute and chronic environmental and pathological stressors, as opposed to “chronological age,” which is simply the passage of time, has become central to this paradigm [1, 2]. The frailty index, which attempts to operationalize this increasing state of vulnerability by adding up a variety of pathological “deficits” across multiple biological and physiological systems [3], has been shown not only to fulfill theoretical assumptions on how damage within a complex biological network might accumulate [4], but also to reliably predict numerous age-related outcomes such as cardiovascular disease risk [5], depression [6], post-operative recovery [7] and death [8, 9]. However, the frailty index has also been criticized with regard to the length of time required to implement, the vast number of deficits required to achieve a reliable score, the use of inherently biased self-reported data and how deficits are treated when deriving a score (i.e., unweighted and often dichotomously) [10].

Another approach, which has received much attention recently, is quantifying biological age using standardized laboratory or other routine clinical measures. Essentially, an increased biological age results from exhibiting levels that are commonly observed for individuals older than one’s self, as in the Klemera–Doubal approach [11], or from exhibiting levels that depart from population averages or established clinical thresholds, as in the homeostatic dysregulation [12] or FI-lab [13] approaches, respectively. Similar to the former is the use of genome-wide DNA (i.e., CpG) methylation data to train mathematical algorithms that attempt to accurately estimate one’s age, under the assumption that age-related changes in the proportion of methylation at a given locus are due to a natural chronological process (i.e., clock) and/or the response to damage that accrues over time [14]. Two of the earliest and more popular examples of this include Horvath’s 353-CpG clock [15] and Hannum’s 71-CpG clock [16], which have been shown to correlate with established health-related risk factors [17, 18] as well as predict all-cause and disease-specific mortality [19]. Newer “second-generation” clocks, such as the Dunedin Pace of Aging Methylation [20], PhenoAge [21] and GrimAge [22], which are trained using a combination of chronological age, health-related risk factors or mortality, have proven to be additionally sensitive to detecting adverse outcomes, namely cardiopulmonary and metabolic disease [22, 23], cancer [24] and death [21, 22].

Surprisingly, only a handful of cross-sectional studies have investigated the association between epigenetically determined age and frailty, most of which only including clocks trained on age [25–29]. While the vast majority of these studies confirm that prevalent frailty is strongly associated with epigenetic age, important questions remain unanswered: First, given that frailty, by definition, is a syndrome encompassing age-related defects in multiple biological and physiological systems that indicate vulnerability to adverse outcomes, would it more strongly associate with clocks that are trained solely on chronological age or health-related risk factors and mortality or a combination of the two? Second, what is the capacity of these contextually different clocks in predicting the change in frailty over time? In the following study, we aimed to investigate the relationships of eight epigenetic clocks with frailty, both cross-sectionally and longitudinally, using baseline and 3-year follow-up data from the Canadian Longitudinal Study on Aging. These clocks were divided into two groups, those trained solely on chronological age and those trained on combination of age, phenotypic markers of health and mortality. The former included clocks developed by Horvath [15], Hannum [16] and Lin [30] as well as a clock by Yang [31], developed to estimate cellular turnover. The latter included four: (1) the Dunedin Pace of Aging Methylation [20], trained on a longitudinal change score of phenotypic and biological measures in relatively young adults; (2) GrimAge [22], based on DNA methylation scores for plasma biomarkers and smoking pack-year exposure, and trained to predict time-to-death; (3) PhenoAge [21], trained on a phenotypic age score known to predict all-cause mortality; and (4) a clock developed by Zhang [32], trained to predict all-cause mortality. We hypothesized that clocks trained on phenotypic markers of health or mortality would best predict changes in the frailty index over time, as compared to clocks trained on chronological age.

## Results

### Summary of cohort sociodemographics and frailty

As summarized in Table 1, the average age of our cohort was 62 years, half were women, and most had post-secondary education, consumed two or more servings of fruits and vegetables per day or reported a total household income of $50,000 or more. Further, nearly half were never smokers and the average physical activity score was 139, which is similar to that previously reported for community-dwelling older adults [33]. At both baseline and at 3-year follow-up, the distribution of the frailty index for the entire cohort was nearly identical: 0.141 ± 0.075 (median [min/max] = 0.127 [0.0132, 0.548]) and 0.142 ± 0.077 (0.129 [0.004, 0.543]), respectively.

**Table 1:** Descriptive summary of participants in the current study, stratified by change in frailty

	Total
	(*N* = 1446)
Age	63 (10.3)
*Sex*	
F	732 (50.6%)
M	714 (49.4%)
*Education*	
Post-secondary	1224 (84.6%)
Secondary	141 (9.8%)
< Secondary	81 (5.6%)
*Income*	
> 100 K	478 (33.1%)
50–100 K	445 (30.8%)
20–50 K	352 (24.3%)
< 20 K	93 (6.4%)
Missing	78 (5.4%)
*Smoking status*	
Never	653 (45.2%)
Former	631 (43.6%)
Current	161 (11.1%)
Missing	1 (0.1%)
*Fruit/veg. consumption*	
4+	783 (54.1%)
2–3	436 (30.2%)
< 2	139 (9.6%)
Missing	88 (6.1%)
Physical activity score	139 (74.5)
Missing	93 (6.4%)
Frailty index (baseline)	0.141 (0.0749)
Missing	3 (0.2%)
Frailty index (3-year)	0.142 (0.0766)
Missing*	179 (12.4%)

Continuous data presented as the average (standard deviation) and categorical data as the count (frequency). * includes participants that did not provide any data at follow-up (*n* = 126), and those in which greater that 10% of frailty index items were missing (*n* = 53)

**Fig. 1:**
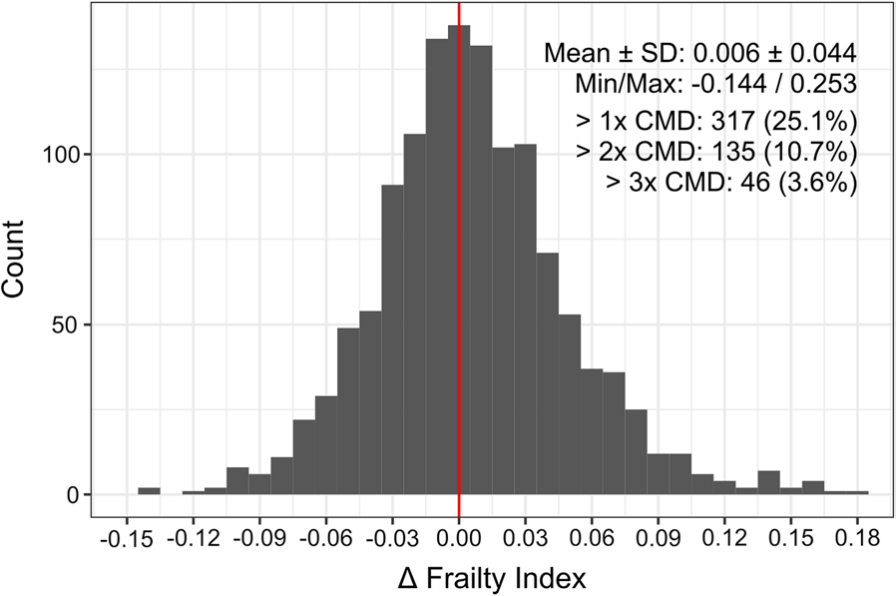
Summary of the change in frailty from baseline to 3-year follow-up. The change in frailty was calculated as the follow-up value minus the baseline value for all participants who provided follow-up data (*n* = 1264). The mean and standard deviation, and minimum and maximum change are shown, along with the number and frequency of participants that exhibited greater than one, two or three times the clinically meaningful difference (CMD) in frailty (i.e., 0.03; also shown as vertical blue lines). The vertical red line shows no difference

For those participants that provided data at both time points (*n* = 1264), an examination of the change in frailty over 3 years (i.e., frailty at follow-up minus baseline) indicated that frailty both increased and decreased (Fig. 1). The average change in frailty was just above zero, 0.006 ± 0.044, and roughly half (54%) of participants exhibited an increase over time; while the proportion was similar between women and men, the average change was greater in women (0.008 ± 0.044 vs. 0.005 ± 0.044, respectively). In terms of a clinically meaningful difference (CMD) in frailty, previously denoted as a change of 0.03 or greater [34, 35], 25% of participants exhibited at least a CMD increase at follow-up, 11% exhibited twice that, and 3.6% exhibited three times the CMD. Alternatively, 18% of participants exhibited at least a CMD decrease at follow-up, and 5% exhibited twice that.

### Characterization of the epigenetic clock measures

**Fig. 2:**
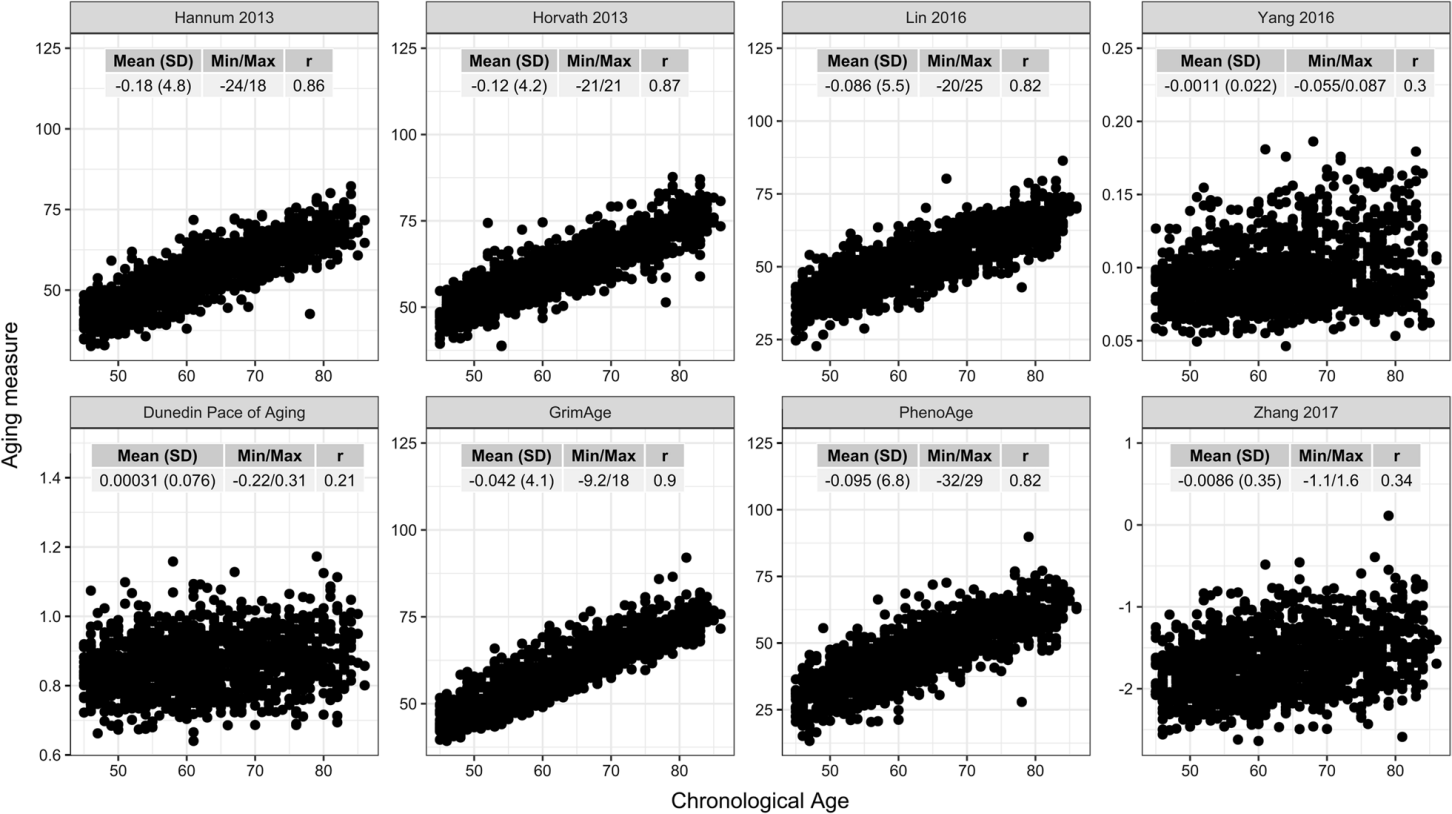
Summary of epigenetic clock measures. In each plot, a respective epigenetic clock estimate (*y*-axis) relative to chronological age (*x*-axis) is presented, along with an inserted table describing the mean (standard deviation) and minimum/maximum for the corresponding delta age estimate. Also shown in each table is the correlation (r) between the epigenetic clock estimate and chronological age

Eight different epigenetic clocks were calculated for the present cohort, many of which differ substantially with regard to the units they are presented in and the variance that they exhibit (Fig. 2; Additional file 1: Table S1). Nonetheless, each correlated significantly (nominal *p* < 0.001) with chronological age, the strongest of which is GrimAge (*r* = 0.90), followed by Horvath [15] (0.87), Hannum [16] (0.86), Lin [30] (0.82), PhenoAge (0.82), Zhang [32] (0.34), Yang [31] (0.30) and Dunedin PoAm (0.21). As expected, delta age estimates for each clock tended to approximate zero and varied between 3 and 5 SDs on either side of the mean. Although the delta age estimate for all clocks was significantly correlated with one another (nominal *p* < 0.001), those clocks that incorporated chronological age during training (i.e., Hannum [16], Horvath [15], Lin [30] and PhenoAge) tended to correlate strongest, as did those clocks that specifically trained on mortality or pace of aging (i.e., Dunedin PoAm, GrimAge, PhenoAge and Zhang [32]); Hannum [16] was unique in that it correlated relatively well with all other clocks, and Dunedin PoAm and Yang [31] was the only pair to be inversely correlated (Additional file 1: Fig. S1).

Similarly shown in recent work by Crimmins and colleagues [36], phenotype- and/or mortality-trained clocks tended to exhibit the strongest associations with sociodemographic and lifestyle factors, the highest being GrimAge, followed by Dunedin PoAm, Zhang [32] and PhenoAge (Additional file 1: Fig. S2). All delta age measures were significantly lower in females as compared to males, with exception to Yang 2016.

### Associations between delta age estimates and frailty

**Fig. 3:**
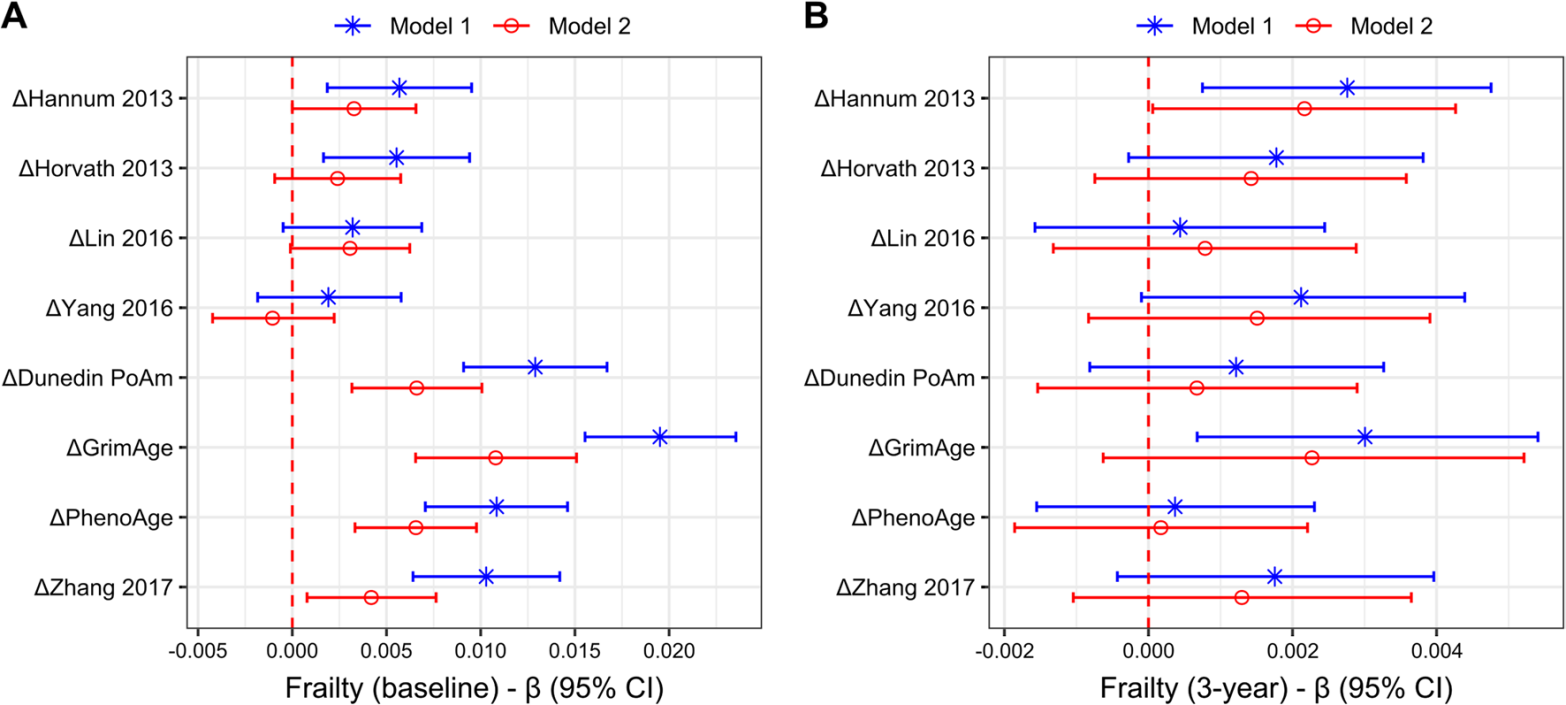
Associations between frailty and different epigenetic clock measures. Frailty at **a** baseline and **b** after 3-year follow-up was regressed on standardized delta age estimates using gamma regression, each of which is in separate models. For both panels, model 1 represents estimates adjusted for age and sex, while model 2 represents estimates adjusted for age, sex, education, income, smoking, diet and physical activity; for **b**, both models were also adjusted for frailty at baseline. Beta coefficients and 95% confidence intervals (CI) are shown, and the dotted red line indicates no association

We first measured the association of each epigenetic clock delta age estimate with frailty at baseline in separate models, adjusting for age and sex (model 1), or age, sex and other sociodemographic factors (model 2), for all participants who provided baseline data (*n* = 1446) (Fig. 3a; Additional file 1: Table S2). In age- and sex-adjusted models, those clocks that were trained on mortality and pace of aging exhibited the strongest associations, led by GrimAge, where frailty increased 0.020 (i.e., 27% of the SD in frailty at baseline for the entire sample) for each 1-SD change in ΔGrimAge (95% CI 0.015, 0.024); the estimates for ΔDunedin PoAm, ΔPhenoAge and ΔZhang 2017 were approximately half of that (standardized beta (95% CI): 0.013 [0.009, 0.017], 0.011 [0.007, 0.015] and 0.010 [0.006, 0.014], respectively). The clocks trained on chronological age and the mitotic clock exhibited weaker associations, and only ΔHannum 2013 (0.0057 [0.0019, 0.0095]) and ΔHorvath 2013 (0.0055 [0.0017, 0.0094]) were significant. When additionally adjusted for sociodemographics, the patterns of estimates remained the same, but were weaker in nearly every case. All mortality-trained clocks and Dunedin PoAm remained significantly associated with baseline frailty, while ΔHannum 2013 and ΔHorvath 2013 failed to retain significance.

**Table 2:** A description of the methodology used to derive each epigenetic clock employed in the current study

Clock	Classification	Normalization approach	CpG availability	Derivation method
Hannum [16]	Age-trained	Noob [57]	65 of 71	R software: ENmix (methyAge function) [64]
Horvath [15]	Age-trained	Noob [57]	334 of 353	Online software: http://dnamage.genetics.ucla.edu/
Lin [30]	Age-trained	Preprocessillumina [57]	97 of 99	Used published weights [30]
Yang [31]	Mitotic clock	BMIQ [65]	354 of 385	R software [31]
Dunedin PoAm [20]	Phenotype	BMIQ [65]	46 of 46	R software: DunedinPoAm38 [20]
GrimAge [22]	Phenotype/mortality	Noob [57]	1030 of 1030*	Online software: http://dnamage.genetics.ucla.edu/
PhenoAge [21]	Phenotype/mortality	Noob [57]	513 of 513	Online software: http://dnamage.genetics.ucla.edu/
Zhang [32]	Mortality	Preprocessillumina [57]	8 of 10	Used published weights [32]

^*^GrimAge CpGs have not been released, so the availability of sites for the current study is assumed given that the algorithm was designed to be compatible with the EPIC 850 K array [22]

To measure the association between delta age estimates and frailty at 3-year follow-up, we used the aforementioned modeling strategy and additionally adjusted for frailty at baseline for all participants that provided data at both time points (*n* = 1246) (Fig. 3b; Additional file 1: Table S2). As with the analysis of frailty at baseline, in age- and sex-adjusted models ΔGrimAge exhibited the strongest association with frailty at follow-up, where for every 1-SD change in ΔGrimAge frailty changed 0.003 (95% CI 0.00068, 0.00541), or approximately 7% of the SD in change in frailty for the entire sample. However, associations with ΔHannum 2013 were nearly as strong (standardized beta (95% CI): 0.0028 [0.00075, 0.00476]) and remained significant in fully adjusted models (0.0022 [0.00006, 0.00426]). We also tested whether the delta age estimates were associated with the likelihood of a CMD increase in frailty at follow-up, but only ΔGrimAge was statistically significant: in age- and sex-adjusted models, for every 1-SD increase, the odds increased by 1.22 times (95% CI 1.09, 1.37), and in models additionally adjusted for sociodemographics, the odds increased by 1.26 times (95% CI 1.09, 1.46) (Additional file 1: Table S2).

## Discussion

The primary goal of the current study was to evaluate a series of conceptually diverse epigenetic clock measures with regard to their association to frailty and its change over 3 years. While the frailty index is an excellent predictor of adverse health outcomes in a variety of settings [5–9], it has also been criticized for being cumbersome and inherently biased [10]; hence, identifying standardized molecular measures that are indicative of its change, especially over relatively short intervals, would be of certain value. In our sample of the CLSA, the change in frailty over 3 years was normally distributed, on average increasing about 20% (i.e., 0.006) of what has been previously described as a clinically meaningful difference (i.e., 0.03) [34, 35]. After adjusting for the minimum age at recruitment, this is similar to what has previously been reported for community samples in the USA, Canada and Europe [37–40].

As hypothesized, clocks that trained on phenotypic markers and/or mortality (i.e., GrimAge, Dunedin PoAm, PhenoAge and Zhang [32]) were most strongly associated with prevalent frailty; this is supported by recently published work [41]. Of those, GrimAge exhibited the strongest association, which is not surprising, as it exhibits robust associations with healthspan and lifespan [22] and specifically incorporates DNA methylation loci that correlate with a number of frailty-related plasma biomarkers, such as leptin [42], TIMP-1 [43], beta-2 microglobulin [44] and cystatin C [45]. Given this, it is also not surprising that GrimAge was significantly associated with the change in frailty over 3 years, which was not observed for the other mortality-trained clocks. It would appear that the unique combination of chronological age and DNA methylation scores of relevant plasma proteins and smoking pack-years provides GrimAge additional sensitivity to detect changes in health that other phenotype or mortality-trained clocks are not afforded.

Among the clocks that were significantly associated with prevalent frailty, Dunedin PoAm was the second highest in magnitude. This is particularly interesting as it attempts to quantify the rate (or pace) at which physiological and phenotypic health-related biomarkers change with age, instead of their levels relative to the population mean or risk of death. The relatively strong association is warranted, especially since our frailty index is predominantly composed of health-related conditions that influence the levels of many of the 18 biomarkers that are part of the Dunedin PoAm, and in a similar direction as chronological age; for example, FEV1 decreases with age and numerous cardiopulmonary disorders [46], while C-reactive protein (CRP) [47] and mean arterial pressure [48] both increase with age and depressive symptoms. Since many of these biomarkers have also been shown to be related to the incidence of frailty-related chronic conditions, it is unclear why the Dunedin PoAm was not significantly associated with the change in frailty. This may have to do with the fact that this clock was trained on the rate of biomarker change in relatively young adults, and may not reflect the “damage” that occurs later in life, which influences the breakdown of biological networks and ultimately determines the trajectory of frailty [49]. Interestingly, the only other clock to be associated with change in frailty was Hannum [16], the coefficient for which was nearly as strong as GrimAge. Like GrimAge [23, 50], Hannum [16] has been shown to be correlated with levels of the chronic inflammatory marker CRP [51, 52], which is significantly related to both prevalent [53] and incident [54, 55] frailty. Another age-trained clock we studied, Horvath [15], was not found to be significantly related to CRP in the same studies as Hannum [16, 51, 52] and was not associated with change in frailty in the current study.

Our study featured both strengths and limitations. Strengths included a relatively large sample of participants derived from the population-based Canadian Longitudinal Study on Aging, from which we derived eight conceptually diverse epigenetic clocks and a comprehensive frailty index based on 76 deficits related to chronic conditions, well-being and physical/cognitive functioning. Furthermore, we were able to investigate frailty longitudinally, which is not common in the literature. Unfortunately, the time between frailty measures was only 3 years, which may not be as reliable a time point to accurately estimate the true trajectory of frailty.

## Conclusions

In summary, we have shown that epigenetic clocks trained on phenotypic markers of health and aging and/or mortality are most strongly associated with prevalent frailty. GrimAge and Hannum [16] were the only clocks to be associated with both baseline and change in frailty, suggesting that they may be most effective at predicting health trajectories of older adults and detecting beneficial effects of healthy aging interventions.

## Methods

### Cohort description

This study was an analysis of data from the Canadian Longitudinal Study on Aging (CLSA) baseline (2012–2015) and first follow-up (2015–2018) collection; the CLSA study design and methods have been previously described [56]. Specifically, it was based on the CLSA comprehensive cohort (baseline dataset version 4.1; follow-up dataset version 3.0), which includes 30,097 community-dwelling adults aged 45–86 years at recruitment who provided questionnaire data through in-home interviews and provided additional physical and cognitive assessment data at one of 11 data collection sites nationwide. Within this cohort, a random pool of 10,000 participants was drawn and extensive laboratory measures, including clinical chemistry and genetics, were performed on cryopreserved blood. From this pool, 1479 participants were randomly selected for DNA methylation analysis on their baseline biospecimen. This study was approved by the Health Sciences North Research Ethics Board (#20-030).

### DNA methylation analysis and description of the final sample

The proportion of methylation on cytosine–guanine (CpG) nucleotide pairs was measured using the Infinium MethylationEPIC BeadChip platform (Illumina, CA, USA) on DNA extracted from peripheral blood mononuclear cells (PBMCs); a summary of this work and the preparation of DNA methylation data can be found at: https://www.clsa-elcv.ca/doc/3491. Briefly, blood was drawn into CPT vacutainers (BD Biosciences, NJ, USA), after which PBMCs were isolated, resuspended in PBS and cryopreserved in vapor-phase liquid nitrogen. From this, DNA was extracted by QIAsymphony nucleic acid extraction platform using DNA midi kits (Qiagen, Hilden, Germany) and bisulfite-treated using the EZ DNA methylation kit (Zymo, CA, USA). Measurement of CpG methylation on converted DNA samples by MethylationEPIC array was performed according to manufacturer’s recommendations. At each step in this process (i.e., DNA extraction, bisulfite conversion and array hybridization and analysis), participant samples were batch-randomized. After the acquisition of raw data, probe-level QC was first performed using functions from the R package “minfi” [57]: The median log intensity of methylated and unmethylated channels was checked using “getQC” and exceeded the recommended threshold of 10.5 for all arrays, while the average probe detection *p*-value (i.e., methylated and unmethylated signals tested against background) for each array, assessed using the “detectionP” function, was at least 0.005. Array-level QC found that of the 1479 samples initially included for DNA methylation analysis, 4 were removed due to poor bisulfite conversion (i.e., < 85%), while another 29 were flagged by built-in outlier detection functions in the R packages “wateRmelon” [58] and “lumi” [59]. Hence, the final sample included 1446 participants. Of those, 1320 provided data at follow-up, while the remaining 126 participants either withdrew from the study (*n* = 53) or died (*n* = 24) prior to providing data, or data were not available for another reason (*n* = 49).

### Derivation of estimates from published epigenetic clocks

Eight epigenetic clocks were chosen based on the phenomenon or outcome they were originally designed to estimate or predict; they are labeled using the name that they are commonly referred to or by the lead author and year of the study in which they were initially published. Horvath [15], Hannum [16] and Lin [30] were trained on chronological age, and therefore, use DNA methylation in order to estimate one’s age. Yang [31] (also known as epiTOC) was also trained on chronological age, but only at CpG sites associated with Polycomb group targets that were also constitutively unmethylated in fetal tissues; based on these criteria, the authors argued that this clock should be highly related to mitotic-like processes. Dunedin Pace of Aging Methylation (PoAm) [20] was trained on a longitudinal change score of 18 biomarkers in adults between the ages of 26 and 38 years. GrimAge [22] was developed using a two-stage process in which DNA methylation scores for 12 age-related plasma biomarkers and smoking pack-year exposure were first identified and then trained to predict time-to-death. PhenoAge [21] was trained on a phenotypic age score based on nine clinically relevant blood biomarkers and chronological age that predicted all-cause mortality, while Zhang [32] was trained on all-cause mortality. Each epigenetic clock was derived using either published software or weights and beta values normalized according to the method that would best recapitulate the authors’ original findings; this, along with the respective number of CpGs used in current study, is found in Table 2. The units for each clock are as follows: Yang 2016 estimates are presented as “pctgAge,” the average methylation level across the sites comprising epiTOC, Dunedin PoAm as years of physiological decline occurring per 12 months of calendar time and Zhang [32] as arbitrary units; all remaining clocks are presented as years. For all clocks, delta age values represent the residual of each respective clock estimate regressed on chronological age.

### Outcomes

Frailty at baseline and 3-year follow-up was estimated using the frailty index approach [3], specifically, 76 deficits related to chronic conditions, activities of daily living, depression, perceptions of health, satisfaction with life, body mass and social participation, as per previous work [60, 61] (Additional file 1: Table S3). It is calculated as the proportion of deficits present relative to the total sum of deficits considered, ranging from 0 to 1, and is gamma distributed [3, 62]; hence, increasing values represent worse health and greater risk of adverse outcomes. As an example, a person reporting ten deficits would exhibit a frailty index of 0.131 (i.e., 10 divided by 76). Frailty was defined as missing for any participant missing more than seven deficit variables (~ 10%).

### Covariates

The following variables were included in regression analysis given that we have previously demonstrated their association to frailty in older adults [60]: age, sex, education, income, smoking, physical activity and diet. Ethnicity was not considered given that only 6% of participants reported being a racial group other than white and even so, only slight differences in the demographic makeup and distribution of epigenetic clock measures were observed between groups (Additional file 1: Table S4). Education was categorized as less than, at least or greater than secondary education. Total household income was defined as annual earnings of less than $20,000, $20,000–50,000, $50,000–100,000 and more than $100,000. Smoking was defined as never (have not smoked 100 cigarettes in their lifetime), former (have smoked at least 100 cigarettes, but have not smoked in the past 30 days) or current (have smoked at least 100 cigarettes and have smoked at least one cigarette in the past 30 days). Physical activity was operationalized using the Physical Activity Scale for the Elderly (PASE) [33], a continuous measure in which a greater score indicates an overall greater amount of time spent per week performing activities such as walking, housework, and sports and recreational activities. Diet was evaluated based on participant fruit and vegetable consumption and defined as less than two servings daily, two–three servings and four or more servings; this information was captured within the AB SCREEN™ II assessment tool (the AB SCREEN™ II assessment tool is owned by Dr. Heather Keller. Use of the AB SCREEN™ II assessment tool was made under license from the University of Guelph). Data for all factors were obtained by a self-reported questionnaire, and refusing or being unable to answer a given question was considered missing.

### Statistical analysis

All continuous sociodemographic variables were summarized as the mean and standard deviation (SD) and categorical variables as the count and percentage. For comparison between groups, either *t*-test or Fisher’s exact test was used, and nominal (unadjusted) *p*-values reported. The association of each epigenetic clock with chronological age (or among epigenetic clocks) was estimated by Pearson’s correlation, while the distribution of each delta age estimate was summarized as the mean, SD, minimum and maximum.

Associations between delta age for each clock and sociodemographic and lifestyle factors were estimated by ordinary least squares regression using two models, the first adjusting for age and sex and the second additionally adjusting for education, income, smoking, physical activity and diet. Given that the frailty index commonly follows a gamma distribution [62], the association between delta age estimates and frailty was estimated by gamma regression (identity link) using the two models as described above. In models with frailty as the dependent variable, all covariates were found to improve model fit statistics (i.e., residual deviance and Akaike's information criterion) and diagnostic criteria (i.e., normality and heteroskedasticity of residuals). For frailty at 3-year follow-up, both models were also adjusted for frailty at baseline in order to determine the association with change in frailty. In all models, delta age was standardized to have a mean of 0 and SD of 1 in order to facilitate cross-clock comparisons. Results are presented as the coefficient (i.e., beta) and 95% confidence interval, which was not adjusted for multiple testing, and any observation including missing data was excluded from analysis; p-values were not reported as confidence intervals tend to provide greater information on the effect size(s) being presented [63]. To estimate the odds of a CMD increase in frailty at follow-up related to each delta age estimate, we used ordinal regression, where the change in frailty was categorized as no increase (i.e., ΔFI ≤ 0), up to 1× CMD (i.e., 0 < ΔFI < 0.03), 1–2× CMD (i.e., 0.03 ≤ ΔFI < 0.06) and 3 or more than 3× CMD (i.e., ΔFI ≥ 0.06). These models were adjusted for age and sex and presented as the odds ratio (OR) and 95% confidence interval (as above, *p*-values are not provided). All analyses were performed in R version 3.6.

## Supplementary Information

**Additional file 1:** Supplementary tables and figures accompanying this manuscript.

## Data Availability

Data are available from the Canadian Longitudinal Study on Aging (www.clsa-elcv.ca) for researchers who meet the criteria for access to de-identified CLSA data.

## References

[1] Jazwinski SM, Kim S (2019). Examination of the dimensions of biological age. Front Genet.

[2] Kennedy BK, Berger SL, Brunet A, Campisi J, Cuervo AM, Epel ES (2014). Geroscience: linking aging to chronic disease. Cell.

[3] Searle SD, Mitnitski A, Gahbauer EA, Gill TM, Rockwood K (2008). A standard procedure for creating a frailty index. BMC Geriatr.

[4] Mitnitski AB, Rutenberg AD, Farrell S, Rockwood K (2017). Aging, frailty and complex networks. Biogerontology.

[5] Wong TY, Massa MS, O’Halloran AM, Kenny RA, Clarke R (2018). Cardiovascular risk factors and frailty in a cross-sectional study of older people: implications for prevention. Age Ageing.

[6] Chu W, Chang S-F, Ho H-Y, Lin H-C (2019). The relationship between depression and frailty in community-dwelling older people: A systematic review and meta-analysis of 84,351 older adults. J Nurs Scholarsh Off Publ Sigma Theta Tau Int Honor Soc Nurs.

[7] Darvall JN, Gregorevic KJ, Story DA, Hubbard RE, Lim WK (2018). Frailty indexes in perioperative and critical care: A systematic review. Arch Gerontol Geriatr.

[8] Zhang X, Dou Q, Zhang W, Wang C, Xie X, Yang Y (2019). Frailty as a predictor of all-cause mortality among older nursing home residents: A systematic review and meta-analysis. J Am Med Dir Assoc.

[9] Kojima G, Iliffe S, Walters K (2018). Frailty index as a predictor of mortality: a systematic review and meta-analysis. Age Ageing.

[10] Chao Y-S, Wu H-C, Wu C-J, Chen W-C (2018). Index or illusion: The case of frailty indices in the Health and Retirement Study. PLOS ONE.

[11] Klemera P, Doubal S (2006). A new approach to the concept and computation of biological age. Mech Ageing Dev.

[12] Li Q, Wang S, Milot E, Bergeron P, Ferrucci L, Fried LP (2015). Homeostatic dysregulation proceeds in parallel in multiple physiological systems. Aging Cell.

[13] Blodgett JM, Theou O, Howlett SE, Wu FCW, Rockwood K (2016). A frailty index based on laboratory deficits in community-dwelling men predicted their risk of adverse health outcomes. Age Ageing.

[14] Field AE, Robertson NA, Wang T, Havas A, Ideker T, Adams PD (2018). DNA methylation clocks in aging: Categories, causes, and consequences. Mol Cell.

[15] Horvath S (2013). DNA methylation age of human tissues and cell types. Genome Biol.

[16] Hannum G, Guinney J, Zhao L, Zhang L, Hughes G, Sadda S (2013). Genome-wide methylation profiles reveal quantitative views of human aging rates. Mol Cell.

[17] Horvath S, Gurven M, Levine ME, Trumble BC, Kaplan H, Allayee H (2016). An epigenetic clock analysis of race/ethnicity, sex, and coronary heart disease. Genome Biol.

[18] Quach A, Levine ME, Tanaka T, Lu AT, Chen BH, Ferrucci L (2017). Epigenetic clock analysis of diet, exercise, education, and lifestyle factors. Aging.

[19] Fransquet PD, Wrigglesworth J, Woods RL, Ernst ME, Ryan J (2019). The epigenetic clock as a predictor of disease and mortality risk: a systematic review and meta-analysis. Clin Epigenetics.

[20] Belsky DW, Caspi A, Arseneault L, Baccarelli A, Corcoran DL, Gao X, et al. Quantification of the pace of biological aging in humans through a blood test, the DunedinPoAm DNA methylation algorithm. Hagg S, Tyler JK, Hagg S, Justice J, Suderman M, editors. eLife. eLife Sciences Publications, Ltd; 2020;9:e54870. 10.7554/eLife.54870 PMC7282814 32367804

[21] Levine ME, Lu AT, Quach A, Chen BH, Assimes TL, Bandinelli S (2018). An epigenetic biomarker of aging for lifespan and healthspan. Aging.

[22] Lu AT, Quach A, Wilson JG, Reiner AP, Aviv A, Raj K (2019). DNA methylation GrimAge strongly predicts lifespan and healthspan. Aging.

[23] Hillary RF, Stevenson AJ, McCartney DL, Campbell A, Walker RM, Howard DM (2020). Epigenetic measures of ageing predict the prevalence and incidence of leading causes of death and disease burden. Clin Epigenetics.

[24] Dugué P-A, Bassett JK, Joo JE, Baglietto L, Jung C-H, Wong EM (2018). Association of DNA methylation-based biological age with health risk factors and overall and cause-specific mortality. Am J Epidemiol.

[25] Breitling LP, Saum K-U, Perna L, Schöttker B, Holleczek B, Brenner H (2016). Frailty is associated with the epigenetic clock but not with telomere length in a German cohort. Clin Epigenetics.

[26] Kim S, Myers L, Wyckoff J, Cherry KE, Jazwinski SM (2017). The frailty index outperforms DNA methylation age and its derivatives as an indicator of biological age. GeroScience.

[27] Zhang Y, Saum K-U, Schöttker B, Holleczek B, Brenner H (2018). Methylomic survival predictors, frailty, and mortality. Aging.

[28] Gale CR, Marioni RE, Harris SE, Starr JM, Deary IJ (2018). DNA methylation and the epigenetic clock in relation to physical frailty in older people: the Lothian Birth Cohort 1936. Clin Epigenetics.

[29] Vetter VM, Spira D, Banszerus VL, Demuth I (2020). Epigenetic clock and leukocyte telomere length are associated with Vitamin D status but not with functional assessments and frailty in the Berlin aging study II. J Gerontol A Biol Sci Med Sci.

[30] Lin Q, Weidner CI, Costa IG, Marioni RE, Ferreira MRP, Deary IJ (2016). DNA methylation levels at individual age-associated CpG sites can be indicative for life expectancy. Aging.

[31] Yang Z, Wong A, Kuh D, Paul DS, Rakyan VK, Leslie RD (2016). Correlation of an epigenetic mitotic clock with cancer risk. Genome Biol.

[32] Zhang Y, Wilson R, Heiss J, Breitling LP, Saum K-U, Schöttker B (2017). DNA methylation signatures in peripheral blood strongly predict all-cause mortality. Nat Commun.

[33] Washburn RA, Smith KW, Jette AM, Janney CA (1993). The physical activity scale for the elderly (PASE): development and evaluation. J Clin Epidemiol.

[34] Theou O, van der Valk AM, Godin J, Andrew MK, McElhaney JE, McNeil SA (2020). Exploring clinically meaningful changes for the frailty index in a longitudinal cohort of hospitalized older patients. J Gerontol A Biol Sci Med Sci.

[35] Jang I-Y, Jung H-W, Lee HY, Park H, Lee E, Kim DH (2020). Evaluation of clinically meaningful changes in measures of frailty. J Gerontol A Biol Sci Med Sci.

[36] Crimmins EM, Thyagarajan B, Levine ME, Weir DR, Faul J. Associations of age, sex, race/ethnicity and education with 13 epigenetic clocks in a nationally representative US sample: The health and retirement study. J Gerontol A Biol Sci Med Sci. 2021. 10.1093/gerona/glab016 PMC8140049 33453106

[37] Chamberlain AM, St Sauver JL, Jacobson DJ, Manemann SM, Fan C, Roger VL (2016). Social and behavioural factors associated with frailty trajectories in a population-based cohort of older adults. BMJ Open.

[38] Mitnitski A, Song X, Rockwood K (2012). Trajectories of changes over twelve years in the health status of Canadians from late middle age. Exp Gerontol.

[39] Stolz E, Mayerl H, Waxenegger A, Rásky É, Freidl W (2017). Impact of socioeconomic position on frailty trajectories in 10 European countries: evidence from the survey of health, ageing and retirement in Europe (2004–2013). J Epidemiol Community Health.

[40] Jadczak AD, Robson L, Cooper T, Bell JS, Visvanathan R (2021). FIRST study collaborators the frailty in residential sector over time (FIRST) study: methods and baseline cohort description. BMC Geriatr..

[41] McCrory C, Fiorito G, Hernandez B, Polidoro S, O’Halloran AM, Hever A, et al. GrimAge outperforms other epigenetic clocks in the prediction of age-related clinical phenotypes and all-cause mortality. J Gerontol A Biol Sci Med Sci. 2020. 10.1093/gerona/glaa286 PMC8087266 33211845

[42] Kane AE, Sinclair DA (2019). Frailty biomarkers in humans and rodents: current approaches and future advances. Mech Ageing Dev.

[43] Jansen HJ, Moghtadaei M, Mackasey M, Rafferty SA, Bogachev O, Sapp JL (2017). Atrial structure, function and arrhythmogenesis in aged and frail mice. Sci Rep.

[44] Liu Z-Y, Shen Y-Y, Ji L-J, Jiang X-Y, Wang X-F, Shi Y (2017). Association between serum β2-microglobulin levels and frailty in an elderly Chinese population: results from RuLAS. Clin Interv Aging.

[45] Hart A, Blackwell TL, Paudel ML, Taylor BC, Orwoll ES, Cawthon PM (2017). Cystatin C and the risk of frailty and mortality in older men. J Gerontol A Biol Sci Med Sci.

[46] McHugh J, Duong M, Ma J, Dales RE, Bassim CW, Verschoor CP (2020). A comprehensive analysis of factors related to lung function in older adults: Cross-sectional findings from the Canadian Longitudinal Study on Aging. Respir Med..

[47] McCaffery JM, Niaura R, Todaro JF, Swan GE, Carmelli D (2003). Depressive symptoms and metabolic risk in adult male twins enrolled in the national heart, lung, and blood institute twin study. Psychosom Med.

[48] Sonsin-Diaz N, Gottesman RF, Fracica E, Walston J, Windham BG, Knopman DS (2020). Chronic systemic inflammation is associated with symptoms of late-life depression: the ARIC study. Am J Geriatr Psychiatry Off J Am Assoc Geriatr Psychiatry.

[49] Rutenberg AD, Mitnitski AB, Farrell SG, Rockwood K (2018). Unifying aging and frailty through complex dynamical networks. Exp Gerontol.

[50] Arpón A, Milagro FI, Santos JL, García-Granero M, Riezu-Boj J-I, Martínez JA. Interaction among sex, aging, and epigenetic processes concerning visceral fat, insulin resistance, and dyslipidaemia. front endocrinol [Internet]. 2019 [cited 2021 Mar 4];10. https://www.ncbi.nlm.nih.gov/pmc/articles/PMC6653993/. 10.3389/fendo.2019.00496 PMC6653993 31379754

[51] Irvin MR, Aslibekyan S, Do A, Zhi D, Hidalgo B, Claas SA, et al. Metabolic and inflammatory biomarkers are associated with epigenetic aging acceleration estimates in the GOLDN study. Clin Epigenetics [Internet]. 2018 [cited 2021 Mar 4];10. https://www.ncbi.nlm.nih.gov/pmc/articles/PMC5907301/. 10.1186/s13148-018-0481-4 PMC5907301 29713391

[52] Stevenson AJ, McCartney DL, Harris SE, Taylor AM, Redmond P, Starr JM (2018). Trajectories of inflammatory biomarkers over the eighth decade and their associations with immune cell profiles and epigenetic ageing. Clin Epigenetics.

[53] Soysal P, Stubbs B, Lucato P, Luchini C, Solmi M, Peluso R (2016). Inflammation and frailty in the elderly: a systematic review and meta-analysis. Ageing Res Rev.

[54] Welstead M, Muniz-Terrera G, Russ TC, Corley J, Taylor AM, Gale CR (2020). Inflammation as a risk factor for the development of frailty in the Lothian Birth Cohort 1936. Exp Gerontol.

[55] Gale CR, Baylis D, Cooper C, Sayer AA (2013). Inflammatory markers and incident frailty in men and women: the English Longitudinal Study of ageing. Age Dordr Neth.

[56] Raina P, Wolfson C, Kirkland S, Griffith LE, Balion C, Cossette B, et al. Cohort profile: The Canadian Longitudinal Study on Aging (CLSA). Int J Epidemiol. 2019;dyz221. 10.1093/ije/dyz173 PMC6929533 31633757

[57] Aryee MJ, Jaffe AE, Corrada-Bravo H, Ladd-Acosta C, Feinberg AP, Hansen KD (2014). Minfi: a flexible and comprehensive Bioconductor package for the analysis of Infinium DNA methylation microarrays. Bioinforma Oxf Engl.

[58] Pidsley R, Wong CCY, Volta M, Lunnon K, Mill J, Schalkwyk LC (2013). A data-driven approach to preprocessing Illumina 450K methylation array data. BMC Genom.

[59] Du P, Kibbe WA, Lin SM (2008). lumi: a pipeline for processing Illumina microarray. Bioinforma Oxf Engl.

[60] Verschoor CP, Tamim H. Frailty is inversely related to age at menopause and elevated in women who have had a hysterectomy: an analysis of the Canadian Longitudinal Study on Aging. J Gerontol A Biol Sci Med Sci. 2018. 10.1093/gerona/gly092 PMC6477649 29688443

[61] Kanters DM, Griffith LE, Hogan DB, Richardson J, Patterson C, Raina P (2017). Assessing the measurement properties of a Frailty Index across the age spectrum in the Canadian Longitudinal Study on Aging. J Epidemiol Community Health.

[62] Rockwood K, Mogilner A, Mitnitski A (2004). Changes with age in the distribution of a frailty index. Mech Ageing Dev.

[63] Rothman KJ (1986). Significance questing. Ann Intern Med.

[64] Xu Z, Niu L, Li L, Taylor JA. ENmix: a novel background correction method for Illumina HumanMethylation450 BeadChip. Nucleic Acids Res. 2016;44:e20. 10.1093/nar/gkv907 PMC4756845 26384415

[65] Tian Y, Morris TJ, Webster AP, Yang Z, Beck S, Feber A (2017). ChAMP: updated methylation analysis pipeline for Illumina BeadChips. Bioinformatics.

