# Supplementary material for: Epigenetic age is associated with baseline and 3-year change in frailty in the Canadian Longitudinal Study on Aging
Source: Clin Epigenetics. 2021 Aug 23;13:163. doi: 10.1186/s13148-021-01150-1 (PMC8381580; doi:10.1186/s13148-021-01150-1)

# Supplementary material

**Supplementary Table 1:** Summary statistics describing estimates from each epigenetic clock measure for the entire cohort.

|  | **Total** |
| --- | --- |
|  | **(N=1446)** |
| **Hannum 2013** |  |
| Mean (SD) | 55.1 (9.24) |
| Median [Min, Max] | 54.4 [32.6, 82.2] |
| **Horvath 2013** |  |
| Mean (SD) | 61.1 (8.50) |
| Median [Min, Max] | 60.7 [38.7, 87.7] |
| **Lin 2016** |  |
| Mean (SD) | 51.7 (9.47) |
| Median [Min, Max] | 51.1 [22.7, 86.4] |
| Missing | 2.00 (0.1%) |
| **Yang 2016** |  |
| Mean (SD) | 0.0944 (0.0228) |
| Median [Min, Max] | 0.0890 [0.0463, 0.186] |
| **Dunedin Pace of Aging** |  |
| Mean (SD) | 0.860 (0.0778) |
| Median [Min, Max] | 0.854 [0.640, 1.17] |
| **GrimAge** |  |
| Mean (SD) | 59.2 (9.20) |
| Median [Min, Max] | 58.6 [39.3, 92.0] |
| **PhenoAge** |  |
| Mean (SD) | 46.2 (11.7) |
| Median [Min, Max] | 45.6 [13.3, 89.8] |
| **Zhang 2017** |  |
| Mean (SD) | -1.68 (0.371) |
| Median [Min, Max] | -1.70 [-2.64, 0.113] |

**Supplementary Table 2:** Estimates from regression modelling of frailty against epigenetic clock measures.

|  | Epigenetic clock | Model 1 | df | Model 2 | df |
| --- | --- | --- | --- | --- | --- |
| Frailty (Baseline)**^a^** | ΔHannum 2013 | **0.006 [0.0019, 0.0095]** | 1439 | 0.003 [-0.0000001, 0.0066] | 1258 |
|  | ΔHorvath 2013 | **0.006 [0.0017, 0.0094]** | 1439 | 0.002 [-0.0009335, 0.0058] | 1258 |
|  | ΔLin 2016 | 0.003 [-0.0005, 0.0069] | 1437 | 0.003 [-0.0001007, 0.0062] | 1257 |
|  | ΔYang 2016 | 0.002 [-0.0018, 0.0058] | 1439 | -0.001 [-0.0042299, 0.0022] | 1258 |
|  | ΔDunedin PoAm | **0.013 [0.0091, 0.0167]** | 1439 | **0.007 [0.0031661, 0.0101]** | 1258 |
|  | ΔGrimAge | **0.02 [0.0155, 0.0235]** | 1439 | **0.011 [0.0065463, 0.0151]** | 1258 |
|  | ΔPhenoAge | **0.011 [0.0071, 0.0146]** | 1439 | **0.007 [0.0033269, 0.0098]** | 1258 |
|  | ΔZhang 2017 | **0.01 [0.0064, 0.0142]** | 1439 | **0.004 [0.000784, 0.0076]** | 1258 |
| Frailty (Follow-up)**^a^** | ΔHannum 2013 | **0.0028 [0.00075, 0.00476]** | 1259 | **0.0022 [0.00006, 0.00426]** | 1150 |
|  | ΔHorvath 2013 | 0.0018 [-0.00028, 0.00381] | 1259 | 0.0014 [-0.00074, 0.00358] | 1150 |
|  | ΔLin 2016 | 0.0004 [-0.00158, 0.00245] | 1257 | 0.0008 [-0.00132, 0.00288] | 1149 |
|  | ΔYang 2016 | 0.0021 [-0.0001, 0.00439] | 1259 | 0.0015 [-0.00083, 0.00391] | 1150 |
|  | ΔDunedin PoAm | 0.0012 [-0.00081, 0.00327] | 1259 | 0.0007 [-0.00154, 0.0029] | 1150 |
|  | ΔGrimAge | **0.003 [0.00068, 0.00541]** | 1259 | 0.0023 [-0.00063, 0.00521] | 1150 |
|  | ΔPhenoAge | 0.0004 [-0.00155, 0.0023] | 1259 | 0.0002 [-0.00186, 0.00221] | 1150 |
|  | ΔZhang 2017 | 0.0018 [-0.00043, 0.00396] | 1259 | 0.0013 [-0.00104, 0.00365] | 1150 |
| Frailty (per CMD)**^b^** | ΔHannum 2013 | 1.092 [0.98, 1.215] | 1257 | 1.07 [0.956, 1.197] | 1148 |
|  | ΔHorvath 2013 | 1.047 [0.939, 1.167] | 1257 | 1.027 [0.916, 1.151] | 1148 |
|  | ΔLin 2016 | 0.956 [0.861, 1.062] | 1255 | 0.958 [0.858, 1.07] | 1147 |
|  | ΔYang 2016 | 1.05 [0.948, 1.164] | 1257 | 1.048 [0.94, 1.169] | 1148 |
|  | ΔDunedin PoAm | 1.082 [0.972, 1.205] | 1257 | 1.068 [0.951, 1.199] | 1148 |
|  | ΔGrimAge | **1.222 [1.087, 1.374]** | **1257** | **1.258 [1.088, 1.456]** | **1148** |
|  | ΔPhenoAge | 1.032 [0.927, 1.149] | 1257 | 1.041 [0.931, 1.165] | 1148 |
|  | ΔZhang 2017 | 1.084 [0.97, 1.211] | 1257 | 1.096 [0.974, 1.235] | 1148 |

For models with frailty at baseline and follow-up as the dependent variables (ie. denoted **^a^**), standardized beta coefficients and 95% confidence intervals from gamma regression are presented for each delta age estimate. For models with the clinically meaningful difference (CMD; no increase, 1xCMD, 2xCMD or 3+CMD) as the dependent variable (ie. denoted **^b^**), standardized odds ratios and 95% confidence intervals from ordinal regression are presented for each delta age estimate. Each delta age estimate was modelled separated and adjusted for age and sex (model 1) or age, sex and sociodemographics (model 2) and results in bold are significant. df, residual degrees of freedom.

**Supplemental Table 3:** Seventy-six variables used to calculate the frailty index.

| **Depressive symptoms (CES-D10 scale) (n=10)** | | **Satisfaction with life (SWLS scale) (n=5)** | |
| --- | --- | --- | --- |
|  | Feeling everything is an effort |  | Life close to ideal |
|  | Feeling depressed |  | Life conditions excellent |
|  | Feeling happy |  | Satisfied with life |
|  | Feeling lonely |  | Have important things in life |
|  | Feeling unable to 'get going' |  | Would change almost nothing about life |
|  | Easily bothered |  |  |
|  | Trouble concentrating | **Activities of daily living (n=14)** | |
|  | Feeling hopeful about the future |  | Able to dress |
|  | Feeling fearful or tearful |  | Able to feed |
|  | Sleep is restless |  | Able to take care of appearance |
|  |  |  | Able to walk |
| **Chronic conditions (n=32)** | |  | Able to get out of bed |
|  | Memory problem |  | Able to take bath |
|  | Dementia or Alzheimer’s disease |  | Trouble to get in time to bathroom |
|  | Epilepsy |  | Able to use telephone |
|  | Migraine headaches |  | Able to travel |
|  | Parkinsonism or Parkinson’s Disease |  | Able to go shopping |
|  | Anxiety disorder |  | Able to prepare meals |
|  | Mood disorder |  | Able to do housework |
|  | Intestinal or stomach ulcers |  | Able to take medicine |
|  | Bowel incontinence |  | Able to handle money |
|  | Urinary incontinence |  |  |
|  | Bowel disorder | **Self-rated health and participation (n=5)** | |
|  | Kidney disease or kidney failure |  | Self-rated general health |
|  | High blood pressure or hypertension |  | Self-rated mental health |
|  | Under-active thyroid gland |  | Self-rated vision |
|  | Over-active thyroid gland (hyperthyroidism) |  | Self-rated hearing |
|  | Diabetes, borderline diabetes or blood sugar is high |  | Health prevents participation in social activities |
|  | Heart disease (including congestive heart failure, or CHF) |  |  |
|  | Peripheral vascular disease or poor circulation in limbs | **Cognitive measures (n=4)** | |
|  | Angina |  | Animal fluency test* |
|  | Stroke or CVA |  | REY I - Immediate Recall* |
|  | Heart attack or myocardial infarction |  | REY II - Delayed Recall* |
|  | Experienced a ministroke or TIA |  | Mental alternation test* |
|  | Back problems excluding fibromyalgia and arthritis |  | * for age, sex and education adjusted t-scores, 0.5-deficit if more than 0.5-SDs below the average and 1.0-deficit if more than 1.5-SDs below the average. |
|  | Arthritis (osteoarthritis or rheumatoid arthritis) |  |  |
|  | Osteoporosis |  |  |
|  | Glaucoma |  |  |
|  | Cataracts | **Physical/performance measures (n=6)** | |
|  | Macular degeneration |  | Body Mass Index (Underweight or Obese as deficit) |
|  | Cancer |  | Average time for 1 chair rise (in seconds)* |
|  | Allergies |  | Average grip strength for all trials* |
|  | Asthma |  | Total time required to complete Timed Get Up and Go (in seconds)* |
|  | Emphysema, chronic bronchitis, COPD, or chronic changes in lungs due to smoking |  | Total time required to complete 4m walk (in seconds)* |
|  |  |  | Best attained time - Standing Balance* |
|  |  |  | * deficit if within the age and sex lower quintile (ie. 20%) |

**Supplementary table 4:** Summary statistics of participant demographics and delta age estimates for each epigenetic clock, stratified by ethnicity.

|  | **Other racial group** | **White** | **Nominal** |
| --- | --- | --- | --- |
|  | **(N=84)** | **(N=1362)** | **p** |
| **Age** | 60.8 (9.27) | 63.1 (10.3) | **0.03** |
| **Sex** |  |  | **0.03** |
| F | 39.0 (46.4%) | 693 (50.9%) |  |
| M | 45.0 (53.6%) | 669 (49.1%) |  |
| **Education** |  |  | 0.75 |
| Post-Secondary | 74.0 (88.1%) | 1150 (84.4%) |  |
| Secondary | 7.00 (8.3%) | 134 (9.8%) |  |
| < Secondary | 3.00 (3.6%) | 78.0 (5.7%) |  |
| **Income** |  |  | 0.65 |
| > 100K | 24.0 (28.6%) | 454 (33.3%) |  |
| 50-100K | 31.0 (36.9%) | 414 (30.4%) |  |
| 20-50K | 22.0 (26.2%) | 330 (24.2%) |  |
| < 20K | 5.00 (6.0%) | 88.0 (6.5%) |  |
| Missing | 2.00 (2.4%) | 76.0 (5.6%) |  |
| **Smoking status** |  |  | 0.67 |
| Never | 42.0 (50.0%) | 611 (44.9%) |  |
| Former | 34.0 (40.5%) | 597 (43.8%) |  |
| Current | 8.00 (9.5%) | 153 (11.2%) |  |
| Missing | 0 (0%) | 1.00 (0.1%) |  |
| **Fruit/Veg. Consumption** |  |  | 0.56 |
| 4+ | 44.0 (52.4%) | 739 (54.3%) |  |
| 3-4 | 26.0 (31.0%) | 410 (30.1%) |  |
| <2 | 11.0 (13.1%) | 128 (9.4%) |  |
| Missing | 3.00 (3.6%) | 85.0 (6.2%) |  |
| **Physical Activity Score** | 156 (105) | 138 (72.1) | 0.14 |
| Missing | 3.00 (3.6%) | 90.0 (6.6%) |  |
| **Frailty Index (baseline)** | 0.137 (0.0708) | 0.142 (0.0752) | 0.52 |
| Missing | 0 (0%) | 3.00 (0.2%) |  |
| **Frailty Index (3-year)** | 0.133 (0.0741) | 0.143 (0.0767) | 0.28 |
| Missing | 5.00 (6.0%) | 174 (12.8%) |  |
| **ΔHannum 2013** | 0.107 (4.12) [0.86] | -0.196 (4.81) [0.86] | 0.52 |
| **ΔHorvath 2013** | 0.444 (3.62) [0.89] | -0.155 (4.2) [0.87] | 0.15 |
| **ΔLin 2016** | -0.107 (5.04) [0.82] | -0.0844 (5.49) [0.82] | 0.97 |
| Missing | 1.00 (1.2%) | 1.00 (0.1%) |  |
| **ΔYang 2016** | -0.00269 (0.0193) [0.14] | -0.00105 (0.0219) [0.3] | 0.46 |
| **ΔDunedin PoAm** | 0.00309 (0.0662) [0.2] | 0.000142 (0.0766) [0.21] | 0.70 |
| **ΔGrimAge** | -0.206 (3.89) [0.89] | -0.0317 (4.09) [0.9] | 0.69 |
| **ΔPhenoAge** | 0.563 (6.72) [0.79] | -0.135 (6.79) [0.82] | 0.36 |
| **ΔZhang 2017** | -0.00699 (0.37) [0.26] | -0.00872 (0.347) [0.35] | 0.97 |

Continuous data summarized as the mean and standard deviation, and categorical as the count and frequency. Data stratified by ethnicity, “other racial group” or “white” (identifying only as white), and differences tested (ie. nominal p) by t-test or Fisher’s exact test. For epigenetic clock estimates, the correlation between the respective DNA methylation age estimate and chronological age, within each strata, is shown in square brackets.

**Supplementary Figure 1:** Correlation matrix of delta epigenetic clock estimates. Significance or correlations determined by Pearson’s correlation test, where: ***, p<0.001; **, p<0.01; and *, p<0.05.


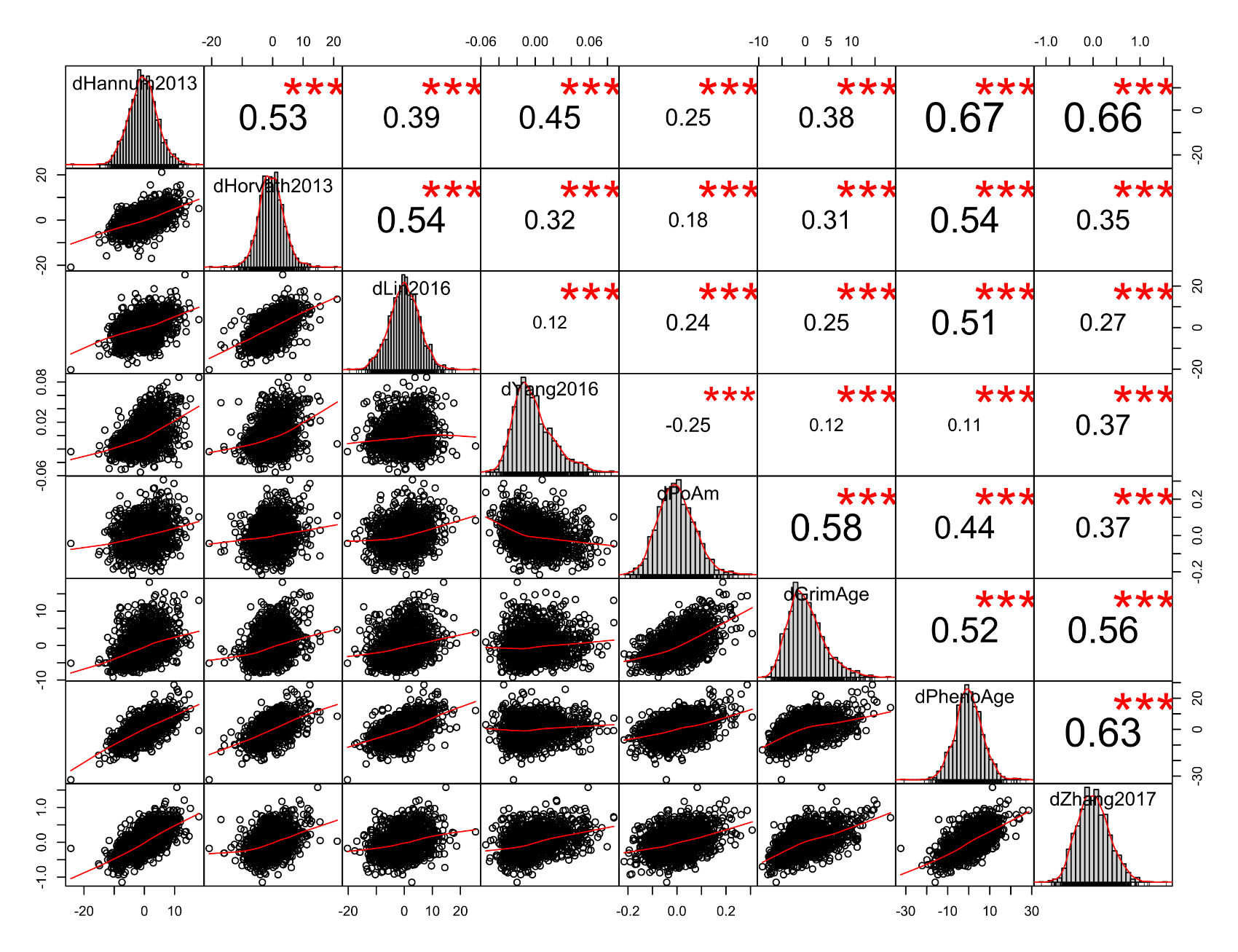


**Supplementary Figure 2:** Associations of sociodemographics and lifestyle factors with epigenetic clocks. Standardized (mean=0, SD=1) delta epigenetic age measures were regressed on sociodemographics and lifestyle factors in A) age and sex adjusted or B) age, sex, education, income, smoking, PASE score, and diet adjusted models. The regression coefficient and 95% confidence interval (CI) is presented relative to the reference category (ref) or per-SD change in the PASE score. Overlap with the red dotted line indicates no significant association.


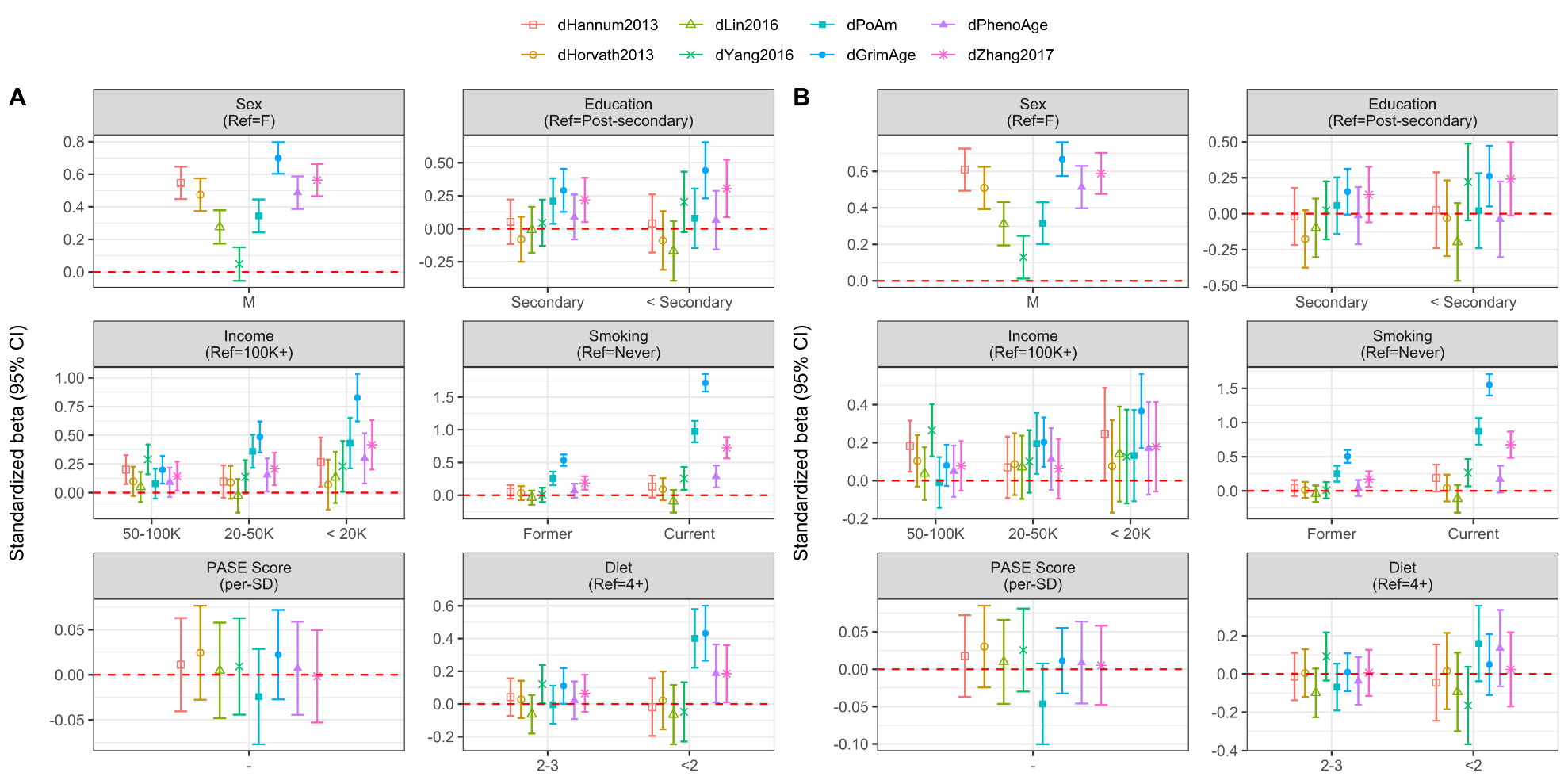

Supplement: Supplementary file 1 — Additional file 1: Supplementary tables and figures accompanying this manuscript. [file 13148_2021_1150_MOESM1_ESM.docx]
